# Special Issue: “Recent Advances in Ion Channels and Ion Channelopathies”

**DOI:** 10.3390/ijms25073598

**Published:** 2024-03-22

**Authors:** Deanne H. Hryciw

**Affiliations:** 1 School of Environment and Science, Griffith University, Nathan, QLD 4111, Australia; d.hryciw@griffith.edu.au; 2 Griffith Institute for Drug Discovery, Griffith University, Nathan, QLD 4111, Australia

The aim of this special issue was to showcase recent advanced in understanding ion channel function and dysfunction associated with disease.

Ion channels play a significant role in normal homeostasis, and contribute to functions required for life such as signal transduction, transepithelial transport, regulation of neurological function and maintenance of cell volume and ion concentrations [1]. The diversity of ion channels contributes to their essential role in the maintenance of life, with researchers often limiting their focus to proteins expressed in humans. It is important to consider that studies focused on ion channels expressed in other animal phylum can inform our understanding between the structure and function of these critical transport proteins.

Chen et al. (contribution 1), investigated the α_2_δ subunit in the high-voltage activated (HVA) calcium channel in the sweet potato rot nematode, *Ditylenchus destructor*. Voltage gated calcium channels are in general classified as low-voltage activated and high-voltage activated based on their electrophysiological properties [2]. The pore forming α_1_ subunit, is supported by the α_2_δ subunit, which is critical for trafficking and glycosylaphosphhatidylinositol dependant activation of the HVA [3]. Chen et al. (contribution 1), demonstrated the voltage-gated calcium channel α2δ subunit (*DdCavα2δ*) in *Ditylenchus destructor* was critical for motility, chemotaxis and reproduction. As *Ditylenchus destructor*, has the potential to harm critically important range of commercial plants including carrots and peanuts, modulation of calcium channel function could be investigated in terms of agricultural benefits.

Ion channelopathies are a heterogeneous group of disorders, caused by ion channel dysfunction due to genetic or acquired factors such as drugs and toxins [4]. The most common cause of ion channelopathies are mutations specifically in genes which encode ion channels (and not accessory proteins), that lead to an impairment of ion channel function [4]. Critical to this space is the ability of a change in genetic sequence in an ion channel to be classified as benign (polymorphic) or pathogenic [5]. 

Neurological ion channels are likely to play a role in homeostatic mechanisms associated with membrane excitability, with ion channelopathies likely to first present in childhood [6]. One disorder, epilepsy, is often characterised by focal and generalised seizures, which may be the result of ion channel dysfunction [7]. Several epilepsy associated genes have been identified in humans, and research by Rychov et al. (contribution 2) and Mazzaferro et al. (contribution 3) demonstrate a role of K+ channel subfamily T member 1 (*KCNT1*) and the α4β2 neuronal nicotinic acetylcholine receptor, respectively in this disorder. Both studies identified specific genetic mutations that lead to an imbalance in neuronal excitability. Critical to this area of research is the use of electrophysiology focused on specific ion channel functioning, stemming from the seminal work from Hodgkin and Huxley [8]. The traditional electrophysiological methods, however, require analysis of mutations individually which results in very low throughput. Recent research in this space has led to the development of high-throughput sequencing and proteomics techniques, which allow for detailed analysis of disease-relevant molecular mutations in ion channels [9], which may improve diagnosis of ion channelopathies associated with genetic mutations.

Targeting ion channel dysfunction in disease is a significant area of research, with traditional drug therapies based off pharmacotherapy. Neuropathic pain is the consequence of lesions to the somatosensory nervous system that alter its structure and function, with pain occurring spontaneously via pathologically amplified activation of the somatosensory nervous system [10]. Natural products have been investigated in the treatment of neuropathic pain as they often have better biocompatibility and fewer adverse side effects [11]. The study by Yang et al. investigated the potential of the natural product Narirutin in reversing neuropathic pain (contribution 4). Narirutin modulates a number of intracellular pathways associated with apoptosis, cell cycle and cell signaling associated with oxidative stress and inflammation [12] To add to this body of research, Yang et al. identified Na_v_1.7 as the molecular target of Narirutin (contribution 4). Research in the field of natural products has the potential to not only identified potential therapeutic drugs, but to characterise the relationship between structure and function of an ion channel.

Cystic fibrosis (CF) is a progressive ion channelopathy caused by mutations in the CF membrane conductance regulator (CFTR) [13]. Much of our understanding of the pathological changes associated with CF has focused on lung dysfunction, however in addition, gastrointestinal dysfunction is also an important pathological feature in CF [14]. Nutritional status, because of gastrointestinal dysfunction, significantly impacts morbidity and mortality [15]. In this issue, research by Shrestha et al. (contribution 5) investigated the essential fatty acid status in a novel rat model of CF. Research in earlier mice models demonstrated that essential fatty acid status in mice models does not always mimic patients with CF [16]. Understanding the ileal genetic changes in essential fatty acid metabolic pathways in the rat model (contribution 5), may provide us with a better understanding about the pathological changes associated with CF in non-lung tissue. Interestingly, a study in the CF pig demonstrated that the essential fatty acid changes are not due to diet, as these changes exist at birth [17]. Thus, in addition to changes in cell signal transduction and transport, ion channel dysfunction may lead to imbalances in lipid metabolism.

In conclusion, ion channels are a diverse group of proteins essential to life, and understanding the relationship between structure and function through investigations in lower order organisms or natural product ligands may provide further insight into this diverse group of proteins. Using both traditional and emerging methods for analysis, the burden of differentiating polymorphism from pathogenetic mutation may be improved. Certainly these studies have contributed to the history of trailblazing theoretical and experimental research focused on ion channels [18], with the adoption of emerging novel technologies likely to contribute to this rapidly evolving field of biology.
